# The role of policies in reducing the cost of capital for offshore wind

**DOI:** 10.1016/j.isci.2023.106945

**Published:** 2023-05-22

**Authors:** Mak Đukan, Anurag Gumber, Florian Egli, Bjarne Steffen

**Affiliations:** 1 Climate Finance and Policy Group, ETH Zürich, Clausiusstrasse 37, 8006 Zürich, Switzerland; 2 Energy and Technology Policy Group, ETH Zürich, Clausiusstrasse 37, 8006 Zürich, Switzerland

**Keywords:** Environmental science, Energy policy

## Abstract

Offshore wind will play a critical role in decarbonizing Europe’s energy infrastructure. Nevertheless, according to recent financing cost surveys, its investment risk expressed as the cost of capital (CoC) is higher than for onshore wind and solar photovoltaics. This perspective elaborates on the possible reasons behind the offshore wind CoC premium and potential remedies. Our analysis discusses that the massive capital expenditures and construction complexity have concentrated European offshore wind ownership among utilities and oil & gas companies that owing to their legacy investments in fossil fuel infrastructure, have higher return expectations for offshore wind assets. Furthermore, these large-scale investors are bidding zero and negative in highly competitive auctions for offshore wind sites, increasing the project’s merchant risks and CoC. We discuss possible policy solutions to alleviate these risks, including revenue stabilization, enabling a more liquid refinancing market, and creating more robust corporate Power Purchase Agreements via government guarantees.

## Highlights

• Cost of capital (CoC) higher for offshore wind than onshore wind and PV in Europe
• Reasons include construction complexity, concentrated ownership, and revenue risks
• Policymakers have various means to address the offshore wind CoC

## Graphical abstract

**Figure:**
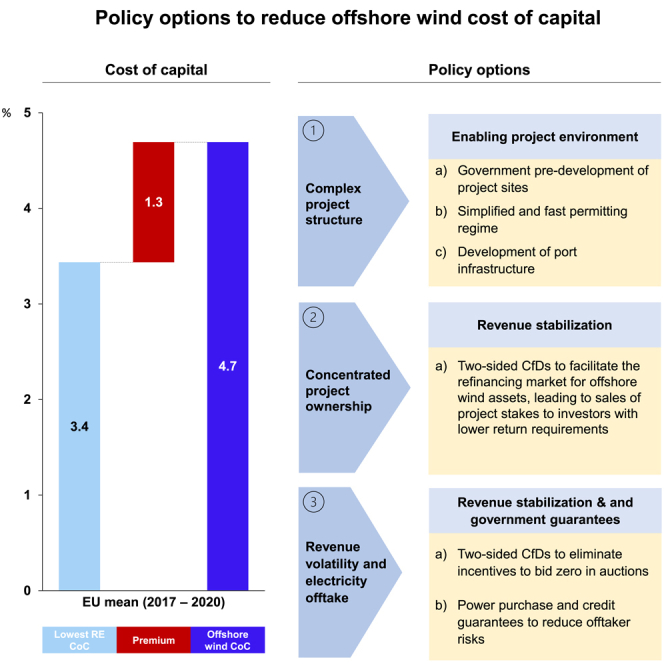

## The offshore wind cost of capital premium

Offshore wind is facing challenges across several fronts, including increasing raw material prices, supply chain bottlenecks, and rising general interest rates.^1^ These changes in the investment environment are delaying projects,^2^ making achieving climate targets more challenging. As part of its European Green Deal, the European Union (EU) plans a 55% CO_2_ emissions reduction by 2030, compared to 1990^,^ and to become climate-neutral by 2050. Reaching these goals will require a massive increase in renewable electricity (RE) investments.^3^ Offshore wind will make up a significant share of these projects, with plans to increase its capacity to 121 GW by 2030^4^^,^^5^ – up from the 28 GW of total installed capacity in 2021.^6^ Policies will be critical in navigating the current investment risks and ensuring offshore wind rollout remains on track.

**Figure 1:**
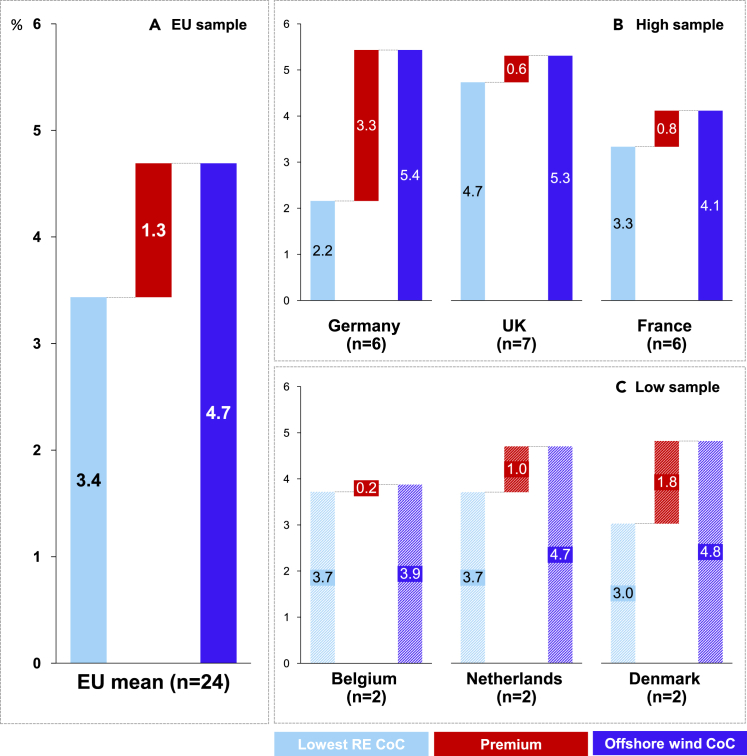
Offshore wind cost of capital premium in Europe (2017–2020) (A) Offshore wind CoC across a sample of EU countries compared to RE technologies with the lowest surveyed CoC in that market. The EU offshore wind premium and the lowest RE CoC is an average of the offshore wind premiums and lowest RE CoC from individual European countries in panels (B) and (C). (B and C) (B) High-sample countries with more than 2 offshore wind CoC estimates (C) Low-sample countries with up to 2 offshore CoC wind estimates. Lowest CoC technologies: PV in Germany and France and onshore wind in the UK, the Netherlands, Belgium, and Denmark. The number of offshore wind estimates: Germany (n = 6), UK (n = 7), Netherlands (n = 2), Belgium (n = 2), Denmark (n = 2), and France (n = 5). Please refer to the Supplementary information for more explanation on the exact method of deriving the presented values. Source of data: Roth et al.^9^ and Taylor et al.^10^

Investors incorporate investment risk in the costs of capital (CoC), representing the expected return capital market participants require to fund a particular investment.^7^ Higher investment risks lead to higher return expectations and CoC.^8^ Figure 1 aggregates for the first time the results of recent RE financing cost surveys^9^^,^^10^ showing offshore wind had, on average, 1.3 percentage points higher CoC than onshore wind and solar photovoltaics (PV) in the leading European offshore wind markets during 2017–2020. In some countries with larger sample sizes, such as Germany (see Figure 1B), the offshore wind CoC premium is even more significant, amounting to 3.3 percentage points, compared to solar PV as the lowest CoC technology in that country. While the size of the CoC premium varies, it is positive in all European countries with offshore wind projects.

**Figure 2:**
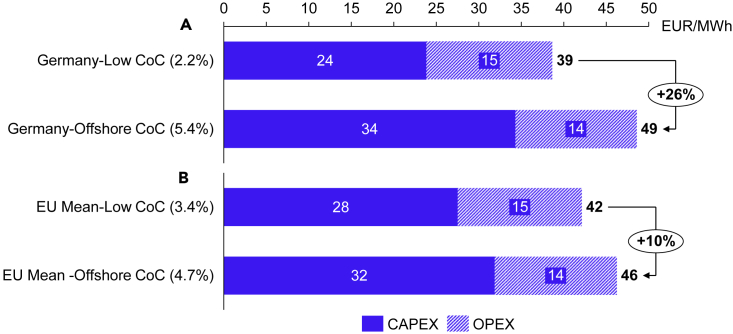
The cost of electricity from offshore wind at different CoC assumptions The levelized cost of electricity (LCOE) for offshore wind at different cost of capital assumptions. (A and B) In panel (A), we show the offshore wind LCOE assuming the lowest recorded CoC for Germany of 2.2% (solar PV) and the German offshore wind CoC of 5.4%. In panel (B), we show the offshore wind LCOE assuming the lowest mean EU-wide CoC consisting of the CoC for PV in Germany and France, and onshore wind in the UK, the Netherlands, Belgium, and Denmark, and the mean EU CoC for offshore wind from the six countries shown in Figure 1. See Supplemental information for cost assumptions and methods.

Previous experiences with onshore wind and solar PV indicate that policy action is critical for de-risking technologies in early deployment phases, reducing the CoC.^11^ Besides accelerating the rollout of offshore wind, decreasing the CoC could also lead to significant reductions in offshore wind production costs, creating less need for public support to make the projects economically viable.^12^ Although the CoC premiums from Figure 1 seem small, they significantly impact electricity production costs.^13^^,^^14^ In a stylized calculation, a 3.3 percentage points CoC premium in Germany leads to 26% higher levelized costs of electricity, as shown in Figure 2A.

To understand the offshore wind CoC premium, in this perspective article, we first elucidate the characteristics of offshore wind investments and how these differ from onshore wind and solar PV. Based on these differences, we consider the possible reasons for the offshore wind CoC premium and the mechanisms that cause it. We then outline policies that could reduce investment risks for offshore wind, providing advice to governments seeking to ensure its steady rollout. This article mainly focuses on Europe – one of the largest global markets for offshore wind with ample available data on RE financing costs. Nevertheless, its findings could apply to other emerging offshore wind markets, such as the US.^15^

## Offshore wind characteristics and their impact on CoC

### Complex project structure

**Figure 3:**
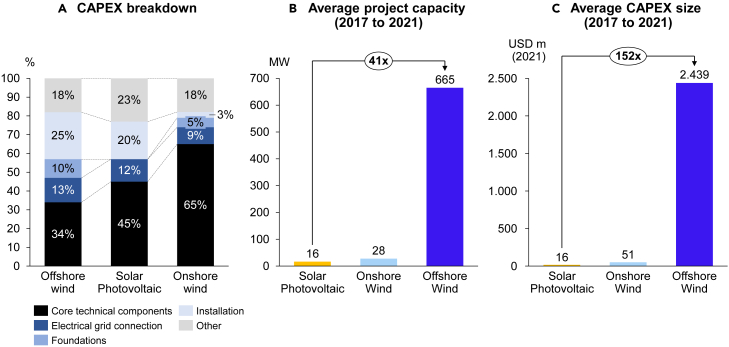
Reasons for complex project structure and concentrated project ownership of offshore wind farms (A) CAPEX breakdown (B) Average project capacity (2017–2021) and (C) Average CAPEX size (2017–2021). (A) Capital expenditures breakdown for offshore wind, utility-scale solar PV, and onshore wind. Core technical components include nacelle, tower, rotor, blades in offshore and onshore wind, modules, inverters, and racking in case of solar PV. Source: Danish Energy Agency Technology Catalog^20^ for offshore wind, IEA^21^ for onshore wind, and solar PV. NREL^22^ data used to derive onshore wind installation costs. (B) Average weighted project capacity (MW) for utility-scale projects (over 1 MW) across Germany, Netherlands, Denmark, the UK, Belgium, and France. Source: Bloomberg New Energy Finance (BNEF).^23^ (C) Average weighted CAPEX size (million USD, 2021) for the same markets and technologies. Source: BNEF.^23^

Offshore wind farms have several characteristics that set them apart from onshore wind and solar PV plants, ultimately impacting their CoC. First, the complexity of developing, constructing, and operating an offshore wind farm is much greater than onshore wind and solar PV plants. Besides the harsher environment at sea, unlike onshore wind and solar PV, offshore wind projects require major additional infrastructure, including underwater substructures and grid infrastructure. To demonstrate the greater project complexity, (in Figure 3A), we break down the capital expenditures (CAPEX) of offshore wind, onshore wind, and solar PV into single categories. In the case of offshore wind, the core technical components comprise only 34% of overall CAPEX, while foundations, grid connection, and installation costs comprise another 48%. In comparison, core technical components account for 45% and 65% of typical utility-scale solar PV and onshore wind CAPEX, respectively. The large shares of grid and foundation costs in the overall CAPEX are a proxy for construction complexity. Offshore wind projects have a complex structure of managerial interfaces with subcontractors hired to develop offshore foundations, the inter-array, and external cables, not to mention harbor management and services.^16^ Hence, there is a higher possibility of cost overruns and cascading delays,^17^^,^^18^ and potential conflicts between project managers and subcontractors during project construction. By extension, the overall investment risk increases, leading to higher CoC. In comparison, the managerial and technical complexity of constructing onshore wind and solar PV projects is lower.

### Concentrated project ownership

Second, offshore wind projects have significantly larger CAPEX amounts than utility-scale solar PV and onshore wind, with an average investment size of 2.4 billion USD compared to 51 and 16 million USD for average onshore wind and utility-scale solar PV projects (Figures 3B and 3C), respectively. The large CAPEX size and construction complexity have concentrated European offshore wind ownership among large-scale utilities and oil & gas companies capable of stomaching the investment sizes and offshore wind construction risks. Unlike onshore wind and solar PV projects with a broader investor pool, utilities like Ørsted (formerly DONG– Danish Oil and Natural Gas), RWE, and Vattenfall, and oil & gas companies such as Equinor (formerly Statoil), Enbridge, and Eni dominate the offshore wind market (see figure in method details). Such large-scale offshore wind investors have the capital to finance projects directly through their balance sheets. However, they also engage in project financing, by funding a separate project company with its separate balance sheet and acquiring debt from banks.^19^

Companies do not always benchmark the profitability of their investments against their CoC. Instead, they frequently use hurdle rates that equal their return expectations,^24^^,^^25^^,^^26^ usually higher than the CoC.^27^^,^^28^ Previous returns often influence current return expectations. For example, the shareholder of companies investing in higher risk and return activities—for instance, in fossil fuel infrastructure—also expect higher returns from their renewable energy investments.^14^^,^^29^^,^^30^ A recent study on CoC finds European utilities with more significant exposure to fossil fuels have greater debt costs and costs of equity. This is due to multiple reasons: climate policies making fossil fuel investments costlier, commodity price swings leading to riskier exploration, and stranded asset risks.^31^ Therefore, the concentration of offshore wind ownership among large-scale utilities and oil & gas companies is one reason to explain the CoC premium for offshore wind. While in the case of balance sheet financing, this materializes in the form of larger return expectations on invested capital; in the case of project financing, it primarily leads to more significant return expectations on the invested equity in the project company.

### Revenue volatility and electricity offtake

Another reason leading to the offshore wind CoC premium is the allocation of support payments via competitive auctions and the specific design of remuneration schemes. Higher shares of RE in the European electricity grids and the new EU State Aid Guidelines for Environmental Protection^32^ ushered the integration of renewables into electricity markets and the allocation of public support via auctions. The combination of competitive auctions and remuneration schemes that connected support payments to electricity prices created additional revenue volatility for onshore and offshore wind and solar energy projects in Europe.^33^^,^^34^ This situation differs from before 2014 when onshore wind and solar PV were technologies with little track record. Policies like the feed-in tariff shielded projects from electricity price risks and guaranteed investors sufficiently high returns.^35^

**Figure 4:**
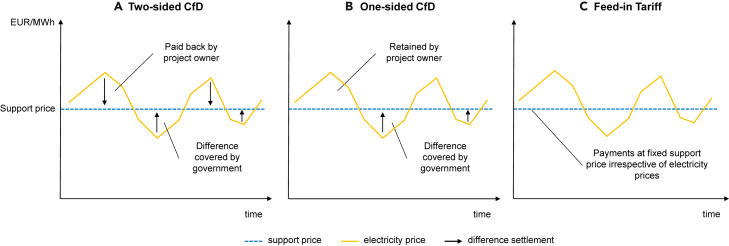
A simplified illustration of how different remuneration schemes function (A) Two-sided CfDs, (B) One-sided CfDs, and (C) Feed-in Tariffs. Image is based on own illustration.

The extent of revenue volatility depends on the design of support remuneration schemes, which are largely the same across the three technologies we assess (except in Denmark; For onshore wind and solar PV projects Denmark applied fixed feed in premiums,^50^ which provide a top-up on the wholesale electricity price). The most applied remuneration schemes in Europe, including the one-sided contract for difference (CfD) in Germany and the Netherlands and the two-sided CfDs like those used in the UK and Denmark, guarantee producers a floor support price equivalent to their bid in the support auction. However, the remuneration schemes differ according to the rules related to excess revenues when the electricity price exceeds the support price. While the one-sided CfDs enable producers to retain excess revenues, the two-sided CfDs mandate producers to pay these revenues back to the government. Figure 4 provides a simplified explanation of how these revenue schemes function. The distinction in remuneration rules leads to a difference in revenue volatility during the support contract.^36^ Put differently, investors can speculate on the upside, i.e., electricity prices above the floor price, when using one-sided CfDs, while two-sided CfDs prevent this incentive.

**Figure 5:**
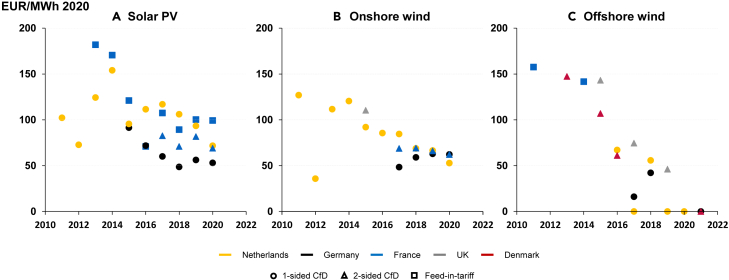
Average awarded auction prices per year (A–C) Average awarded auction prices per year from 2010 to 2022 in EUR/MWh for (A) solar PV (B) onshore wind and (C) offshore wind. Sources: for onshore wind and solar PV auctions, we use the AURES2 auction database^50^ and specifically the column Adjusted average awarded price [ct_2019/kWh]. We use the study by Jansen et al. (2022)^6^ and the column Winning bid, EUR2020/MWh (own calculation) for offshore wind auction data. To make the graph more readable, we deleted the average value for solar PV in France in 2012, which amounted to 266 EUR/MWh. We adjust the AURES2 prices to 2020 values using a 2% inflation rate for all the countries. We do not chart the auctions for Danish fixed premiums for onshore wind and solar PV as these are not directly comparable to the other auction awards (a fixed premium is an add-on to the electricity price).

Allowing upside revenue retention enabled bidders in auctions for one-sided CfDs to bid the lowest possible amount or zero. A 0 EUR/MWh bid means the bidder assumes the complete market risk at the time of bidding; provided there is no long-term price-risk mitigation, the project’s price volatility would resemble those of a merchant power plant. From 2017 to 2021, the German government awarded 2,768 MW (The 2017 and 2018 average awarded auction prices for offshore wind in Germany in Figure 5 are higher than zero. In 2017, Gode Wind 3 (110 MW) was awarded an auction price of 62 EUR/MWh and in 2018 Baltic Eagle (476 MW) and Gode Wind 4 (131 MW) were awarded prices of 68 and 101 EUR/MWh in 2018, respectively. The remaining auctioned projects in 2017 and 2018 for which awarded prices are known were awarded zero bids.^37^) of the 4,058 MW of auctioned offshore wind sites to bidders with zero bids.^37^ Furthermore, driven by the rapid drop in bid prices for auctioned sites in 2016, the Dutch government decided to organize auctions that allowed “zero bids” only.^37^ As of 2017, bidders competed for the auctioned sites based on qualitative criteria such as “the knowledge and experience of the parties involved”, “contribution to the ecology of the North Sea”, and others.^37^^,^^38^^,^^39^ Until 2020, the Netherlands auctioned 3,755 MW or almost all offshore wind sites without price hedging through CfDs.^37^^,^^39^ While onshore wind and solar PV projects were also subject to auctions and the same remuneration schemes, Figure 5 shows that the average awarded auction prices remained between 63 and 75 EUR/MWh on average between 2017 and 2021 for the two technologies in the European markets with one-sided CfDs, respectively.

In principle, one-sided CfDs can also stabilize revenues provided they guarantee a high-enough floor support price. However, there are many reasons why auctions for one-sided CfDs failed to achieve this, leading to zero bids for offshore wind. First, the auction designs directly impact this development, for instance, the decision of the Dutch government to allow only zero-bid auctions and instead select winners based on qualitative criteria. Another auction design example is the 2021 auction for the 800–1000 MW Thor offshore wind farm in Denmark^40^ resulting in several zero bids and a lottery draw to decide on the winning bidder.^41^ Although the auction was for a two-sided CfD, the Danish Energy Agency designed the auction with a clause stipulating maximum CfD payments from the winning bidder to the government. After reaching the maximum amount of 2.9 billion DKK or 390 million EUR (expressed in 2021 prices), the bidder has no more obligations to pay the government,^42^ and in effect, the scheme turns from a two-sided to a one-sided CfD. Therefore, Thor bidders speculated on the potential earnings they could achieve by selling electricity outside the government-backed remuneration scheme. In connection with this, unlike onshore wind and solar PV auctions that call bidders to compete for projects in rounds—where each round is expressed in the volume of installed capacity—offshore wind auctions are typically single-item, meaning bidders compete for single sites. The growing interest in offshore wind from well-capitalized utilities and oil & gas companies meant fierce competition for the sites.

Second, project sponsors expect an increase in wholesale electricity prices in the coming decades and significant future cost reductions with larger turbine sizes in the coming years, leading to lower production costs. Third, zero bids have a real-option component because of the long timelines between the auction award and the project realization.^43^ Among the countries we assess, successful offshore wind bidders reached, on average, their Final Investment Decision (FID) 25 months (This excludes France, where projects needed an average of 100 months to reach FID. However, this was due to permitting issues that led to substantial projects delays. Hence, we exclude France from analyzing average months between an auction award date and FID date.) after the auction award date,^37^ meaning they had time to reassess the market and their financing arrangements and cancel the awarded contract. The non-realization penalties for Germany’s first successful zero-bid projects amounted to between 2.5% and 3.8% of total project development costs.^43^ Hence, the bidders faced high potential earnings and a relatively smaller downside of paying the penalty. Finally, it is important to note that bidding zero did not mean the projects were subsidy-free, as the German and Dutch governments paid for the grid connection and site assessment costs.^37^ Also, for “zero bids”, the German and the Dutch governments still transferred significant public funds into connecting the projects to the grid and paying for a substantial amount of site development.

The effects of zero bids on CoC are 2-fold. First, project sponsors compensate for the higher price risk by increasing their cost of capital or hurdle rates, compared to investing in the same project with stable revenues.^44^^,^^45^^,^^46^^,^^47^ Figure 1 implies that Germany—which implements a one-sided CfD—has a significantly larger offshore wind CoC premium than France and UK, where investors compete in auctions for two-sided CfDs.^6^ However, earlier interview-based research finds fierce competition for single sites could also have caused investors to decrease their hurdle rates to win the auction.^33^ Future research should focus on disentangling the impacts of these two contradictory effects on the CoC for offshore wind. Second, to make their projects bankable, zero bids can lead sponsors to arrange alternative revenue stabilization before reaching an FID. These involve signing corporate power purchase agreements (PPAs) with companies having an investment-grade credit rating (From Aaa to Baa3 for Moody’s, AAA to BBB- for Standard & Poor’s and AAA to BBB1 for Fitch^78^) and significant long-term demand for electricity^47^ – such as Amazon, Google, and Facebook—the top three off-takers of renewable electricity worldwide in 2020.^48^ Such arrangements enable the projects to reach financial close through project financing, the dominant way of financing offshore wind assets in Europe.^19^ However, the financing conditions for projects backed with corporate PPAs greatly depend on the volume of project electricity production contracted under the PPA and the credit-worthiness of the off-taker.

Corporate PPA contracts with highly rated off-takers, long duration, and price hedge for high shares of contracted electricity volume in the project’s overall production have the most positive effect on financing. However, they are still worse than having government-backed remuneration. According to recent survey data (from Australia, but still relevant for this discussion), renewable energy projects with greater exposure to corporate off-takers and merchant risks tend to have higher credit spreads and lower debt shares than CfDs.^49^ Moreover, banks typically mandate loan repayment periods equaling the duration of PPAs.^11^ Recent corporate PPAs with offshore wind farms usually have a duration of 10–15 years (for example see references^79^^,^^80^^,^^81^), shorter than government-backed remuneration schemes that typically last 15 to 20 years.^50^ Hence, corporate PPAs decrease the time span projects sponsors have to repay loans, which under equal debt structuring (Debt may be structured in different forms – as fixed annuity payments of interest and principal, as sculpted payment to match cash flows of the project, or as balloon payment at the end of debt repayment period, to provide relief from paying principal in the early years. In effect, a balloon repayment with a debt repayment of 10 years might lead to same debt sizing as other debt repayment schedules with a longer time to maturity.) will lead to lower debt-to-equity ratios.^51^ Overall, the impacts of zero bids and the greater reliance of project sponsors to mitigate risks through the private sector result in higher CoC, contributing to the offshore wind CoC premium.

More recently, offshore wind investors have started to signal they are willing to pay negative bids to obtain rights to develop projects. In 2021, the UK government held its Offshore Wind Leasing Round 4, collecting option fees worth 879 million GBP annually for rights to develop offshore wind sites until the second auction phase for CfD contracts.^52^^,^^53^ Furthermore, for its Hollandse Kust West auction in 2022, the Dutch government based 10% of its auction scoring criteria on a one-off bidder payment for the rights to develop the project, amounting to a maximum of 50 million EUR.^54^ Similarly, as of 2023, the German government will organize its first auctions requiring bidders to make an unlimited one-off payment for site development rights.^55^ So why does having a higher CoC matter if investors are still willing to submit zero and negative bids and take the risk?

### Why does having a higher CoC matter?

Financial markets place a price on uncertainty. Investors buy assets with more volatile prices, expecting a higher return.^56^ Accordingly, offshore wind projects with more volatile revenues will find an investor operating with a higher CoC. However, in most instances, the sponsors of projects with zero (and negative) bids will seek corporate PPAs to mitigate the price-risk exposure. The question looming over the offshore wind industry is: are there enough corporate buyers with a high-enough credit rating and long-term demand for electricity to hedge price risks for the ever-growing number of zero and negative offshore wind projects?

The counterparty risk significantly impacts project bankability. Counterparties with a higher default probability and revenue volatility increase risk, leading banks that finance offshore wind assets to demand more stringent loan repayment conditions, such as imposing higher Debt Service Coverage Ratios.^57^ Furthermore, corporate PPAs have different structures, varying the price risk they impose on the renewable electricity producer and the corporate buyer.^58^ Those PPA structures that impose higher price risk on the producer might result in more frequent financial distress during the loan repayment, such as violation of debt covenants or inability to service debt. Because of this situation, investors bidding zero and negative in auctions will have higher CoC—and thus ultimately higher production cost—compared to a situation where they had a (non-zero) government-backed PPA. This effect matters because higher offshore wind production cost will ultimately affect overall electricity costs to society (at least as long as the public purse still covers some costs, such as grid connection). Offshore wind is infrastructure critical to successfully achieving the energy transition, and project failures due to higher risk exposure would delay reaching EU offshore wind goals.

**Figure 6:**
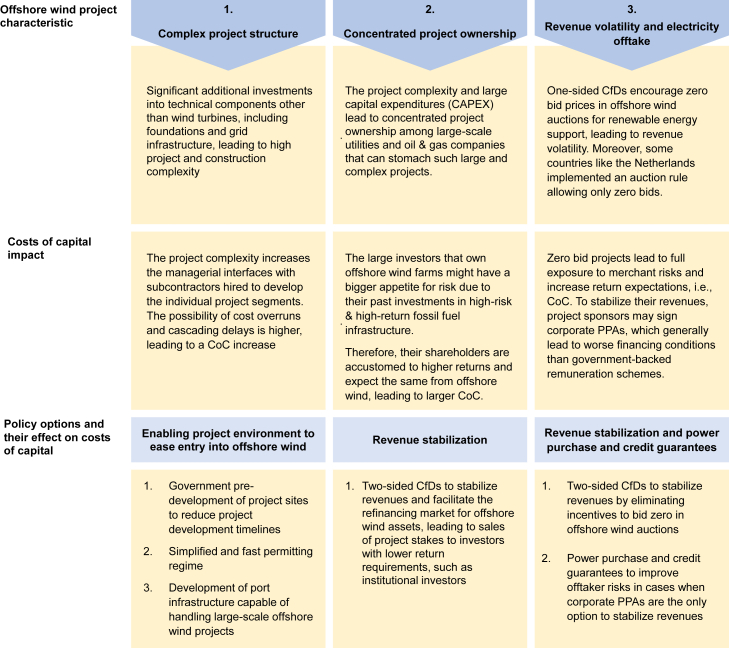
Summary of offshore wind project characteristics, their impacts on costs of capital, and policy options to reduce the CoC premium

However, public policy has the means to address the CoC premium. Figure 6 summarizes the offshore wind project characteristics leading to the CoC premium and the policy options that could help decrease offshore wind risks and their CoC. We discuss these policy measures in the next section.

## Policy options to reduce the CoC premium

### Revenue stabilization via two-sided CfDs

Many policy experts and industry practitioners agree that stabilizing revenues is most effective in reducing the CoC of renewable energy projects. Both one-sided and two-sided CfDs can stabilize revenues, with two-sided CfDs being more favorable for debt financing, as argued previously, by industry and academia.^12^^,^^16^^,^^36^^,^^44^^,^^46^^,^^49^^,^^59^ The optimal CfD designs regarding hedging, the production volume, the reference period duration, etc., are the subject of discussion.^59^^,^^60^ However, the current debate lacks input on how revenue stabilization impacts offshore wind transactions and how this ultimately leads to lower CoC.

First, the CoC is a dynamic value that changes during the project’s lifetime. As the projects advance, transitioning from development and construction to operation, their risks decrease, and so do return expectations from investors.^16^ The change in the assets risk profile allows project sponsors developing and constructing the project to refinance—i.e., sell project stakes to investors with lower return requirements or negotiate better terms with lenders. CfDs with a floor price that secures most project revenues facilitate this process by guaranteeing long-term price stability. For example, to recycle capital back into the company and reduce the project CoC, Ørsted pioneered so-called “farm downs” early on. These involve selling a minority stake to investors with lower return requirements,^62^ for instance, its 258 MW Burbo Bank offshore wind farm (with two-sided CfD from the UK).^61^ Such investors include pension funds, insurance companies, or corporations seeking to green their electricity production.

In contrast, merchant projects without government-backed remuneration demonstrate how the lack of government risk hedging transfers the project’s value to corporates willing to hedge price risks. An example is the Netherlands' Hollandse Kust Zuid project. Vattenfall won the different project segments with zero bids^6^ and sold a 49.5% stake to BASF at almost no profit, or for 2.16 million EUR/MW,^63^ roughly the same as average offshore wind project costs in 2020.^20^ In turn, BASF signed a corporate PPA stabilizing the project’s revenues.^63^ The corporate PPA increased the project’s value. Consequently, three months later, BASF sold half of its shares to Allianz^64^ for an undisclosed amount but at a significant profit margin, according to unofficial sources.^65^

If the Dutch government applied a two-sided CfD, Vattenfall could have anticipated the project sale and submitted a lower auction bid.^16^ Under the current electricity prices, the outcome could resemble UK auctions for two-sided CfDs where investors bid below long-term electricity price expectations, leading them to make difference payments to the government. The project would benefit public finances similar to one-sided CfDs with negative bids. On top, the public would also directly reap the benefits of stabilizing revenues.

Therefore, European policymakers governing Europe’s main offshore wind markets should consider CfDs as mechanisms with two critical roles. First, CfDs transfer price-risk hedging to stable Western European states. Second, they facilitate project re-financing and sales, helping broaden the offshore wind project ownership structure to investors with lower CoC such as pension funds and insurance companies.^17^ Third, they reduce the CoC, and in case of bids well above current electricity prices, they decrease public support costs.^16^

### Power purchase and credit guarantees

Governments could undertake several other measures for projects that are not de-risked via governmental revenue stabilization but sell their electricity to commercial customers. One option is to provide credit guarantees to banks issuing loans to projects singing a PPA with a corporate counterparty with a lower credit rating or no official rating from a rating agency.^66^^,^^67^To our knowledge, Norway’s Power Purchase Guarantee Scheme is currently the only such scheme in Europe. Under the scheme, Norway’s export credit agency Eksfin guarantees sellers of renewable electricity a maximum of 80% of outstanding financial obligations from corporate buyers from Norway’s wood processing, metal production, and chemical industries.^68^ For example, under the backing of the scheme, the Green Investment Group—a UK-based green infrastructure investor—recently signed 18-year PPAs for its two Norwegian onshore wind farms (126.8 MW) with Norway’s steel producer Eramet,^69^^,^^70^^,^^71^ a company not rated by any rating agency.^72^ The guarantee reduces the risk of the corporate off-takers' inability to pay the purchased electricity due to events like financial insolvency. The Danish export credit agency EKF also provides guarantees, however, not for corporate power purchases but for commercial loans given to renewable energy investors using Danish technology, for instance, wind turbines. Schemes that place the weight of governments with solid credit ratings behind offshore wind investments could help developers with zero bids finance their investments with more favorable terms.

### Enabling project environment

In addition to addressing revenue and off-taker risks, governments could tackle other well-documented investment barriers. As we pointed out, developing offshore wind projects is more complex than realizing onshore wind and solar PV. Efficient permitting procedures and stable regulatory frameworks would ease project development and, in the long term, lead to CoC reductions through financial learning or the accumulation of experience with evaluating and financing projects.^11^ The Danish and Dutch governments are leading the way by pre-developing sites, while Germany will follow suit as of 2023 with part of its auctioned offshore wind capacity.^73^ However, in other markets, there is still significant room for improvement. For instance, the UK government announced plans to reduce the consent time for offshore wind from the current four to one year.^5^

Emerging offshore wind markets outside Europe are a case in point regarding the importance of efficient permitting. For instance, the USA plans to build 30 GW of offshore wind until 2030.^74^ However, currently, it has only 42 MW of installed capacity, partly because of its complex permitting regime and changes in federal governments that put issued permits into question.^75^ Furthermore, a thriving offshore wind industry requires a developed port infrastructure that minimizes construction, operation, and maintenance risks. Actions like these could create a more accessible entry point for investors into offshore wind and facilitate learning across the technology and financing value chains.

Altogether, the measures outlined in Figure 6 could help de-risk offshore wind projects and reduce their CoC. Notably, the effect of such public risk mitigation strategies on the overall public cost of offshore wind deployment depends on the policy design. Reducing price risks via two-sided CfDs could stabilize project revenues and, combined with other de-risking measures such as efficient permitting, lead to lower auction prices and support costs. However, in the case of one-sided CfD auctions, risk mitigation strategies, such as guarantees and the enabling environment (see above), could increase negative bids, for instance, under the current German auction designs. While this would benefit the public coffers in the short term, it could cause problems if the projects experience financial distress in the long run due to higher exposure to price risks. Hence, policymakers should calibrate de-risking policies carefully to achieve a fast rollout of offshore wind in Europe while ensuring the lowest possible public cost and long-term financial stability. Smart policy design can contribute to keeping offshore wind deployment on track with climate targets in the EU and beyond.

### Limitations of the study

This study has several limitations. First, CoC is confidential data that is hard to come by. To our knowledge, we build on the most extensive available datasets for offshore wind CoC, which, however, have relatively few data inputs per technology and country (also due to the person-hour effort for personal interviews needed to gather these sensitive data). For further details on the methods used to collect the CoC values, the reader should refer to the study by Roth at al. (2021),^9^ describing the steps in detail. Similar methods were used to obtain the data from the study by Taylor et al. (2023).^10^ We use the Taylor et al. (2023)^10^ data with permission from the authors. Second, offshore wind is a new technology with fewer project examples per country and policy design. Therefore, conclusions regarding policy effectiveness are difficult. Third, our study mainly investigates Europe. We do not analyze other major markets, such as China and USA, which have different CoC values, and whose local context leads to different conclusions regarding policies that decrease CoC.

## STAR★Methods

### Key resources table

**Table:** 

REAGENT or RESOURCE	SOURCE	IDENTIFIER
**Deposited data**		

The cost of capital of onshore wind, solar PV and offshore wind (1)	Roth et al.^9^	https://open-research-europe.ec.europa.eu/articles/1-136/v2
The cost of capital of onshore wind, solar PV and offshore wind (2)	Taylor et al.^10^	https://www.irena.org/Publications/2023/May/The-cost-of-financing-for-renewable-power
Average project capacity, average CAPEX size	BNEF – Asset Finance^23^	https://about.bnef.com/
Offshore wind CAPEX breakdown	DEA^20^	https://ens.dk/en/our-services/projections-and-models/technology-data
Onshore wind and solar PV CAPEX breakdown	IEA^21^	https://www.iea.org/articles/what-is-the-impact-of-increasing-commodity-and-energy-prices-on-solar-pv-wind-and-biofuels
Onshore wind and solar PV auction results	AURES II^50^	http://aures2project.eu/auction-database/
Offshore wind auction results	Jansen et al.^6^	https://www.sciencedirect.com/science/article/pii/S0301421522002257

**Software and algorithms**		

Excel	Microsoft	https://www.microsoft.com/en-us/microsoft-365/excel
R software	Bell Laboratories	https://www.r-project.org/

### Resource availability

#### Lead contact

Further information and requests should be directed to and will be fulfilled by the lead contact, Mak Đukan (mak.dukan@gess.ethz.ch).

#### Materials availability

This study did not generate new materials.

### Method details

#### Figure 1

We merged the data from two financing cost surveys.^9^^,^^10^ For data from Roth et al. (2021),^9^ we used the full survey dataset and not the averaged values available in the online depository Zenodo. Readers of iScience can access the full dataset by contacting the original data provider Eclareon. Regarding the Taylor et al. (2023)^10^ data, the readers can access aggregated values in the report appendix. For obtaining access to the entire dataset, the readers should contact the authors.

We calculate the offshore wind CoC premium in Figure 1 using (Equation 1), (Equation 2), (Equation 3), (Equation 4), (Equation 5):

(Equation 1) $${CoC}_{tech/country-i}=\sum_{2017}^{2021}{CoC}_{\frac{tech}{country}-i}/\;N_{\frac{tech}{country}-i}$$

(Equation 2) $${CoC}_{offshore\;p/country-i}={CoC}_{offshore/country-i}-\min\left({CoC}_{onshore/country-i},{CoC}_{solar/country-i}\right)$$

(Equation 3) $$\bar{C}oC_{offshore/EU}=\sum {CoC}_{offshore/Country-i}/\;N_{countries}$$

(Equation 4) $$\bar{C}oC_{\min tech/EU}=\sum \min\left({CoC}_{onshore/Country-i},{CoC}_{solar/Country-i}\right)/\;N_{countries}$$

(Equation 5) $$\bar{C}oC_{offshore\;p/EU}=\bar{C}oC_{offshore/EU}-\bar{C}oC_{\min tech/EU}$$

where ${CoC}_{tech/country-i}$ is the average cost of capital per technology and country i, $\sum_{2017}^{2021}{CoC}_{tech/country-i}$ is the sum of CoC values per technology and country i, $N_{tech/country-i}$ is the number of survey inputs for each technology and country i, ${CoC}_{offshore\;p/country-i}$ is the offshore wind premium per country i, $\bar{C}oC_{offshore/EU}$ is the average offshore wind cost of capital for the selected EU countries, $\bar{C}oC_{\min tech/EU}$ is the average of the average minimum CoC per technology and country and the $\bar{C}oC_{offshore\;p/EU}$ is the offshore wind CoC premium on the EU level.

#### Figure 2

We calculate the LCOE following Egli et al. (2018),^11^ and as shown in Equation 1:

(Equation 6) $$LCOE=\frac{{CAPEX}_{it}}{\sum_{r=1}^{r=27}\frac{{FLH}_{ity}}{{\left(1+{CoC}_{i}\right)}^{y}}}+\frac{\sum_{r=1}^{r=27}\frac{{OPEX}_{ity}}{{\left(1+{CoC}_{i}\right)}^{y}}}{\sum_{r=1}^{r=27}\frac{{FLH}_{ity}}{{\left(1+{CoC}_{i}\right)}^{y}}}$$

where ${CAPEX}_{it}$ is the initial capital expenditure, ${FLH}_{ity}$ are the full-load hours in years y, the ${CoC}_{i}$ are the costs of capital values from Figure 1, and the ${OPEX}_{ity}$ are the operational expenditures in years y. For calculating the LCOE, we use the investment data for offshore wind turbines for 2020 provided by the Danish Energy Agency (DEA)^15^ including CAPEX and fixed OPEX values for 2020, an operational lifetime of 27 years and full load hours equaling 4.400 MWh/MW.^20^ Furthermore, we apply an inflation index to the OPEX values, considering a 2% inflation rate during the project's lifetime.

#### Figure 3A

Regarding Figure 3A, we assume the cost structure for offshore wind turbines from the DEA^20^ (page 245). Regarding onshore wind and solar PV, we take the cost structure from the International Energy Agency (IEA).^21^ We do not assume DEA data for onshore wind and solar PV because 1) the DEA data on solar PV does not include a separate category for racking. In our analysis, we compare the share of core technical components between the technologies, including nacelle, tower, rotor, and blades for offshore and onshore wind and modules, inverters, and racking in the case of solar PV. Therefore, assuming DEA data would make comparisons between solar PV and other technologies incomparable 2) the DEA divides onshore wind turbine costs into equipment, installation, decommissioning, grid connection etc. Like solar PV, this makes the direct comparison with offshore wind impossible – specifically concerning foundation costs.

On the other hand, assuming IEA data enables direct comparisons between the three technologies, with one exception, which we adjust for. The IEA data does not explicitly include installation costs. We assume a 3.1% installation costs share from NREL^76^ and deduct this from the IEA cost categories “other” – 15% and “freight” – 6% to arrive at the 18% “other” cost category shown in Figure 3A.

#### Figure 3B

We calculate the average project capacity using the reported transaction capacity in the asset finance database from Bloomberg New Energy Finance^23^ for newly built assets only. The projects are filtered for those that had secured financing, were under construction, were commissioned (fully or partially), or had operation or construction suspended i.e., financing was arranged for them. For offshore wind, we exclude floating offshore plants which are currently in the demonstration phase. Further, only projects where disclosed transaction values exist are evaluated. The average project capacity for offshore wind, onshore wind, and solar PV is thereafter calculated by averaging the reported capacities in Belgium, Denmark, France, Germany, Netherlands and UK, between 2017 and 2021.

#### Figure 3C

We calculate the average CAPEX size using the reported transaction value in US dollar in the asset finance database from Bloomberg New Energy Finance^23^ for newly built assets only. Like Figure 3B the projects are filtered for those that had secured financing, were under construction, were commissioned (fully or partially), or had operation or construction suspended i.e., financing was arranged for them. For offshore wind, we exclude floating offshore plants which are currently in the demonstration phase. The average CAPEX size for offshore wind, onshore wind, and solar PV are calculated by averaging the reported transaction values in Belgium, Denmark, France, Germany, Netherlands, United Kingdom, between 2017 and 2021, albeit adjusted for inflation. The transaction values are adjusted to 2021 levels using consumer price inflation data sourced from the World Bank for individual countries.^77^

#### Figure 5

We calculate the onshore wind and solar PV average auction results based on the AURES2 auctions database^50^ specifically, the column “Adjusted average awarded price [ct_2019 / kWh]”. Regarding offshore wind auctions, we use the data from Jansen et al. (2022)^6^ containing a more comprehensive database of offshore wind auction results. We convert the AURES 2 auction results from 2019 to 2020 values using a 2% inflation rate to make the auction results comparable. The values shown in Figure 5 are average auction results per technology, year, country, and remuneration scheme. We conduct the calculations in Excel.

#### Figure 7

We calculate the number of projects a firm has participated in using the reported project ownership data in the renewable project database from Bloomberg New Energy Finance^23^ for newly built assets only. The projects are filtered for those that had secured financing, were under construction, were commissioned (fully or partially), or had operation or construction suspended i.e., financing had been arranged. For offshore wind, we exclude floating offshore plants which are currently in the demonstration phase. The number of projects is thereafter calculated by summing the reported projects in Belgium, Denmark, France, Germany, Netherlands and UK, prior to 2021. The classification of firms is conducted using industry classification obtained from Bloomberg terminal under the Bloomberg Industry Classification System. The classification is thereafter supplemented with manual online inspection of the websites of 147 companies which own offshore wind assets.

**Figure:**
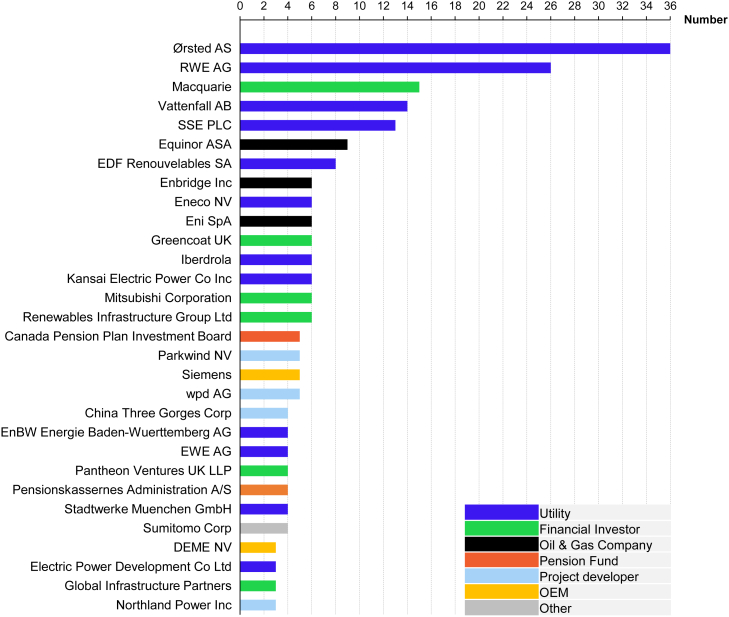
Count of ownership in projects with Final Investment Decision (FID) in the major European markets, including Germany, the UK, Netherlands, Belgium, Denmark, and France OEM refers to original equipment manufacturers. Source: BNEF.^23^
